# *N*-(2-Nitro­oxyeth­yl)picolinamide

**DOI:** 10.1107/S1600536811039572

**Published:** 2011-09-30

**Authors:** Drielly A. Paixão, Silvana Guilardi, Ângelo de Fátima, Débora P. Araujo, Francinely C. Oliveira

**Affiliations:** a Instituto de Química – UFU, Uberlândia, MG, Brazil; b Departamento de Química – UFMG, Belo Horizonte, MG, Brazil

## Abstract

In the title mol­ecule, C_8_H_9_N_3_O_4_, the amide group is involved in the formation of an intra­molecular N—H⋯N hydrogen bond. In the crystal, mol­ecules related by translation along the *a* axis are linked into chains *via* weak inter­molecular C—H⋯O inter­actions.

## Related literature

For related structures, see: Eremenko *et al.* (1996 ▶); Fedorov *et al.* (2001 ▶). For further synthetic details, see: Samejima (1960 ▶); Jiao *et al.* (1990 ▶).
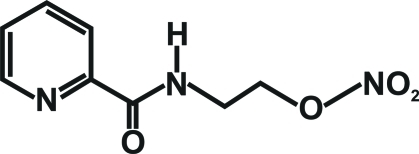


## Experimental

### 

#### Crystal data

C_8_H_9_N_3_O_4_
*M* *_r_* = 211.18
Orthorhombic, 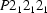

*a* = 5.5075 (2) Å
*b* = 13.6114 (5) Å
*c* = 12.6822 (4) Å
*V* = 950.72 (6) Å^3^
*Z* = 4
Mo *K*α radiation
μ = 0.12 mm^−1^
*T* = 295 K
0.49 × 0.21 × 0.19 mm

#### Data collection

Nonius KappaCCD diffractometer
4037 measured reflections
1265 independent reflections
1039 reflections with *I* > 2σ(*I*)
*R*_int_ = 0.020

#### Refinement

*R*[*F* ^2^ > 2σ(*F* ^2^)] = 0.038
*wR*(*F* ^2^) = 0.108
*S* = 1.07
1265 reflections
137 parameters
H-atom parameters constrained
Δρ_max_ = 0.23 e Å^−3^
Δρ_min_ = −0.15 e Å^−3^

### 

Data collection: *COLLECT* (Nonius, 2000 ▶); cell refinement: *DENZO*/*SCALEPACK* (Otwinowski & Minor, 1997 ▶); data reduction: *DENZO*/*SCALEPACK*; program(s) used to solve structure: *SHELXS97* (Sheldrick, 2008 ▶); program(s) used to refine structure: *SHELXL97* (Sheldrick, 2008 ▶); molecular graphics: *ORTEP-3 for Windows* (Farrugia, 1997 ▶); software used to prepare material for publication: *WinGX* (Farrugia, 1999 ▶).

## Supplementary Material

Crystal structure: contains datablock(s) I, global. DOI: 10.1107/S1600536811039572/cv5154sup1.cif

Structure factors: contains datablock(s) I. DOI: 10.1107/S1600536811039572/cv5154Isup2.hkl

Supplementary material file. DOI: 10.1107/S1600536811039572/cv5154Isup3.cml

Additional supplementary materials:  crystallographic information; 3D view; checkCIF report

## Figures and Tables

**Table 1:** Hydrogen-bond geometry (Å, °)

*D*—H⋯*A*	*D*—H	H⋯*A*	*D*⋯*A*	*D*—H⋯*A*
N2—H⋯N1	0.86	2.31	2.692 (3)	107
C8—H8*B*⋯O1^i^	0.97	2.39	3.239 (3)	145

Symmetry code: (i) 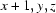.

**Fig. 1.**
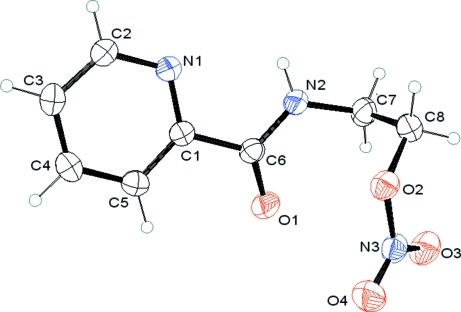
The molecular structure of (I) showing the atom labeling. Displacement ellipsoids are drawn at the 30% probability level.

## supplementary crystallographic information

### Comment

The title compound (I) can be considered as a potential nitric oxide donating drug. Herewith we present its crystal structure.

The molecule of (I) adopts a folded conformation and contains a planar pyridine cycle (NC_5_H_4_) bearing a CO group attached to the α-carbon atom (Fig. 1). The dihedral angle between the pyridine ring and the O1/C6/N2 plane is 11.3 (2)°. This angle is smaller than that in the nicorandyl [22.8 (2)°] (Eremenko *et al.*, 1996).The dihedral angle between the mean planes O1/C6/N2/C7 and C8/O2/N3/O3/O4 is 44.74 (7)°. The C═O and CH_2_–ONO_2_ bonds are oriented *trans* to the pyridine nitrogen atom. In the nicorandyl compound these groups were found in the *cis* position (Eremenko *et al.*, 1996). Another structural isomer (Fedorov *et al.*, 2001), [*N*-(2-nitrooxyethyl)isonicotinamide], displays a different molecular conformation and crystallizes in the same centrosymmetric space group *P*2_1_/*c* as nicorandil.

The crystal structure of (I) is stabilized through weak non-classical intermolecular H-bonds of the type C—H···O in [100] direction, involving the carbon atom of the nitrooxyethyl group and the oxygen atom of carbonylamide. Moreover, were observed one intramolecular interactions of the type N—H···N (Table 1). On the other hand, the compound nicorandil has only one intermolecular interaction of the type N—H···O. The results for compound (I) and its structural isomers show that the position of the ligand in pyridine ring affects the conformation of the molecule and the interactions present in the crystal packing.

### Experimental

The title product was synthesized by heating ethylnicotinate with an excess 2-ethanolamine to give *N*-(2-hydroxyethyl)picolinamide in 92% yield (Samejima, 1960). The nitration of *N*-(2-hydroxyethyl)picolinamide (0.10 mmol) was held by mixing it with fuming nitric acid (1.00 mmol) at -5°C and stirred for 2 h.

The reaction mixture was poured into water and ice, and the pH was adjusted to 6.0 adding (CaCO_3_). The white solid obtained was filtered at reduced pressure and recrystallized in ethanol, forming the *N*-(2-nitrooxyethyl)picolinamide in 63% yield (Jiao *et al.*, 1990). MP: 61.2–63.0°C. ^1^H-NMR (200 MHz, CDCl_3_): 3.85 (2*H*, q, J = 5.6 Hz), 4.67 (2*H*, t, J = 5.2 Hz),7.42–7.48 (^1^H, m), 7.83–7.90 (^1^H, m), 8.19 (^1^H, d, J = 7.9 Hz), 8.39 (^1^H, br s), 8.56 (^1^H, d, J = 4.4 Hz). ^13^C-NMR (200 MHz, CDCl_3_): 36.7, 71.7, 122.2, 126.4, 137.4, 148.1, 149.11, 164.7.

### Refinement

H atoms were geometrically positioned (C—H 0.93-0.97 Å, N—H 0.86 Å) and refined as riding, with U_iso_(H) = 1.2 U_eq_ of the parent atom. In the absence of significant anomalous scatterers in the molecule, attempts to confirm the absolute structure by refinement of the Flack parameter in the presence of 871 sets of Friedel equivalents led to an inconclusive value of -0.2 (13). Therefore, the Friedel pairs were merged before the final refinement.

### Crystal data

**Table:**

C_8_H_9_N_3_O_4_	*D*_x_ = 1.475 Mg m^−^^3^
*M**_r_* = 211.18	Mo *K*α radiation, λ = 0.71073 Å
Orthorhombic, *P*2_1_2_1_2_1_	Cell parameters from 2165 reflections
*a* = 5.5075 (2) Å	θ = 2.9–27.5°
*b* = 13.6114 (5) Å	µ = 0.12 mm^−^^1^
*c* = 12.6822 (4) Å	*T* = 295 K
*V* = 950.72 (6) Å^3^	Prism, colourless
*Z* = 4	0.49 × 0.21 × 0.19 mm
*F*(000) = 440	

### Data collection

**Table:**

Nonius KappaCCD diffractometer	1039 reflections with *I* > 2σ(*I*)
Radiation source: Enraf–Nonius	*R*_int_ = 0.020
graphite	θ_max_ = 27.5°, θ_min_ = 3.2°
Detector resolution: 9 pixels mm^-1^	*h* = −7→7
CCD rotation images, thick slices scans	*k* = −16→17
4037 measured reflections	*l* = −15→15
1265 independent reflections	

### Refinement

**Table:**

Refinement on *F*^2^	Secondary atom site location: difference Fourier map
Least-squares matrix: full	Hydrogen site location: inferred from neighbouring sites
*R*[*F*^2^ > 2σ(*F*^2^)] = 0.038	H-atom parameters constrained
*wR*(*F*^2^) = 0.108	*w* = 1/[σ^2^(*F*_o_^2^) + (0.0654*P*)^2^ + 0.0645*P*] where *P* = (*F*_o_^2^ + 2*F*_c_^2^)/3
*S* = 1.07	(Δ/σ)_max_ < 0.001
1265 reflections	Δρ_max_ = 0.23 e Å^−^^3^
137 parameters	Δρ_min_ = −0.15 e Å^−^^3^
0 restraints	Extinction correction: *SHELXL*
Primary atom site location: structure-invariant direct methods	Extinction coefficient: 0.124 (13)

### Special details

**Table:**

Geometry. All e.s.d.'s (except the e.s.d. in the dihedral angle between two l.s. planes) are estimated using the full covariance matrix. The cell e.s.d.'s are taken into account individually in the estimation of e.s.d.'s in distances, angles and torsion angles; correlations between e.s.d.'s in cell parameters are only used when they are defined by crystal symmetry. An approximate (isotropic) treatment of cell e.s.d.'s is used for estimating e.s.d.'s involving l.s. planes.
Refinement. Refinement of *F*^2^ against ALL reflections. The weighted *R*-factor *wR* and goodness of fit *S* are based on *F*^2^, conventional *R*-factors *R* are based on *F*, with *F* set to zero for negative *F*^2^. The threshold expression of *F*^2^ > σ(*F*^2^) is used only for calculating *R*-factors(gt) *etc*. and is not relevant to the choice of reflections for refinement. *R*-factors based on *F*^2^ are statistically about twice as large as those based on *F*, and *R*- factors based on ALL data will be even larger.

### Fractional atomic coordinates and isotropic or equivalent isotropic displacement parameters (Å^2^)

**Table:**

	*x*	*y*	*z*	*U*_iso_*/*U*_eq_	
O1	−0.1820 (3)	0.49141 (13)	0.05722 (14)	0.0662 (5)	
O2	0.2098 (3)	0.55263 (11)	−0.11459 (12)	0.0528 (4)	
O3	−0.0499 (4)	0.67762 (12)	−0.11167 (15)	0.0705 (5)	
O4	−0.1235 (4)	0.54631 (16)	−0.20249 (14)	0.0797 (6)	
N1	0.1892 (3)	0.31135 (13)	0.17680 (14)	0.0509 (5)	
N2	0.2136 (4)	0.49676 (13)	0.10379 (15)	0.0532 (5)	
H	0.3331	0.4648	0.1308	0.064*	
N3	−0.0061 (4)	0.59684 (13)	−0.14445 (15)	0.0532 (5)	
C1	−0.0042 (4)	0.34497 (13)	0.12528 (15)	0.0430 (5)	
C2	0.1867 (5)	0.21623 (15)	0.20374 (19)	0.0570 (6)	
H2	0.3191	0.1914	0.2407	0.068*	
C3	−0.0002 (5)	0.15319 (15)	0.17994 (18)	0.0565 (6)	
H3	0.0066	0.0875	0.1998	0.068*	
C4	−0.1974 (5)	0.18928 (16)	0.12612 (19)	0.0561 (6)	
H4	−0.3265	0.1484	0.1086	0.067*	
C5	−0.2003 (4)	0.28722 (15)	0.09855 (18)	0.0516 (5)	
H5	−0.332	0.3138	0.0626	0.062*	
C6	−0.0002 (4)	0.45146 (14)	0.09262 (15)	0.0459 (5)	
C7	0.2509 (5)	0.59775 (15)	0.07192 (18)	0.0592 (6)	
H7A	0.3618	0.629	0.1209	0.071*	
H7B	0.0973	0.6325	0.0754	0.071*	
C8	0.3520 (5)	0.60631 (17)	−0.03806 (19)	0.0595 (6)	
H8A	0.3569	0.6751	−0.0581	0.071*	
H8B	0.5171	0.5816	−0.0387	0.071*	

### Atomic displacement parameters (Å^2^)

**Table:**

	*U*^11^	*U*^22^	*U*^33^	*U*^12^	*U*^13^	*U*^23^
O1	0.0545 (10)	0.0624 (10)	0.0817 (12)	0.0089 (8)	−0.0073 (9)	0.0174 (9)
O2	0.0556 (9)	0.0472 (7)	0.0555 (8)	0.0094 (7)	0.0037 (7)	0.0021 (6)
O3	0.0751 (12)	0.0563 (9)	0.0800 (12)	0.0189 (9)	0.0063 (10)	0.0087 (9)
O4	0.0799 (13)	0.0921 (13)	0.0672 (11)	−0.0228 (11)	−0.0137 (10)	0.0037 (10)
N1	0.0505 (10)	0.0471 (9)	0.0550 (10)	−0.0004 (8)	−0.0099 (9)	0.0039 (8)
N2	0.0585 (12)	0.0429 (8)	0.0581 (11)	−0.0035 (8)	−0.0129 (9)	0.0069 (8)
N3	0.0538 (11)	0.0526 (10)	0.0533 (11)	0.0014 (9)	0.0050 (9)	0.0093 (8)
C1	0.0447 (11)	0.0457 (10)	0.0385 (10)	0.0022 (9)	0.0016 (9)	−0.0010 (7)
C2	0.0584 (13)	0.0492 (11)	0.0633 (14)	0.0021 (10)	−0.0085 (12)	0.0074 (10)
C3	0.0685 (15)	0.0433 (10)	0.0577 (13)	−0.0026 (11)	0.0050 (12)	0.0021 (9)
C4	0.0574 (13)	0.0521 (12)	0.0588 (12)	−0.0105 (11)	0.0019 (11)	−0.0068 (10)
C5	0.0455 (11)	0.0559 (12)	0.0534 (12)	−0.0002 (9)	−0.0029 (10)	−0.0011 (9)
C6	0.0491 (12)	0.0459 (9)	0.0428 (10)	0.0022 (9)	−0.0026 (9)	0.0003 (8)
C7	0.0773 (17)	0.0433 (10)	0.0570 (13)	−0.0099 (11)	−0.0131 (12)	0.0011 (9)
C8	0.0519 (13)	0.0536 (12)	0.0728 (16)	−0.0090 (10)	−0.0059 (12)	0.0097 (11)

### Geometric parameters (Å, °)

**Table:**

O1—C6	1.225 (3)	C2—C3	1.374 (3)
O2—N3	1.385 (3)	C2—H2	0.93
O2—C8	1.445 (3)	C3—C4	1.374 (4)
O3—N3	1.200 (2)	C3—H3	0.93
O4—N3	1.197 (3)	C4—C5	1.378 (3)
N1—C1	1.330 (3)	C4—H4	0.93
N1—C2	1.339 (3)	C5—H5	0.93
N2—C6	1.337 (3)	C7—C8	1.506 (3)
N2—C7	1.447 (3)	C7—H7A	0.97
N2—H	0.86	C7—H7B	0.97
C1—C5	1.378 (3)	C8—H8A	0.97
C1—C6	1.508 (3)	C8—H8B	0.97
			
N3—O2—C8	115.41 (16)	C5—C4—H4	120.6
C1—N1—C2	116.73 (19)	C4—C5—C1	118.7 (2)
C6—N2—C7	122.2 (2)	C4—C5—H5	120.7
C6—N2—H	118.9	C1—C5—H5	120.7
C7—N2—H	118.9	O1—C6—N2	123.66 (18)
O4—N3—O3	129.1 (2)	O1—C6—C1	121.03 (18)
O4—N3—O2	112.47 (19)	N2—C6—C1	115.30 (18)
O3—N3—O2	118.4 (2)	N2—C7—C8	112.60 (19)
N1—C1—C5	123.50 (19)	N2—C7—H7A	109.1
N1—C1—C6	116.99 (18)	C8—C7—H7A	109.1
C5—C1—C6	119.49 (19)	N2—C7—H7B	109.1
N1—C2—C3	123.7 (2)	C8—C7—H7B	109.1
N1—C2—H2	118.1	H7A—C7—H7B	107.8
C3—C2—H2	118.1	O2—C8—C7	112.49 (19)
C4—C3—C2	118.6 (2)	O2—C8—H8A	109.1
C4—C3—H3	120.7	C7—C8—H8A	109.1
C2—C3—H3	120.7	O2—C8—H8B	109.1
C3—C4—C5	118.8 (2)	C7—C8—H8B	109.1
C3—C4—H4	120.6	H8A—C8—H8B	107.8
			
C8—O2—N3—O4	−175.41 (19)	C7—N2—C6—O1	−1.7 (3)
C8—O2—N3—O3	5.3 (3)	C7—N2—C6—C1	177.33 (19)
C2—N1—C1—C5	−0.5 (3)	N1—C1—C6—O1	−170.5 (2)
C2—N1—C1—C6	−178.70 (19)	C5—C1—C6—O1	11.2 (3)
C1—N1—C2—C3	0.9 (3)	N1—C1—C6—N2	10.4 (3)
N1—C2—C3—C4	−0.5 (4)	C5—C1—C6—N2	−167.8 (2)
C2—C3—C4—C5	−0.3 (3)	C6—N2—C7—C8	−94.3 (3)
C3—C4—C5—C1	0.6 (3)	N3—O2—C8—C7	76.5 (2)
N1—C1—C5—C4	−0.2 (3)	N2—C7—C8—O2	52.7 (3)
C6—C1—C5—C4	177.95 (19)		

### Hydrogen-bond geometry (Å, °)

**Table:**

*D*—H···*A*	*D*—H	H···*A*	*D*···*A*	*D*—H···*A*
N2—H···N1	0.86	2.31	2.692 (3)	107.
C8—H8B···O1^i^	0.97	2.39	3.239 (3)	145.

Symmetry codes:  (i) *x*+1, *y*, *z*.

## References

[bb1] Eremenko, I. L., Golubnichaya, M. A., Nefedov, S. E., Baranovskyii, I. B., Ol‘shnitskaya, I. A., Ellert, O. G., Novotortsev, V. M., Eremenko, L. T. & Nesterenko, D. A. (1996). *Russ. J. Inorg. Chem.* **41**, 1924–1938.

[bb2] Farrugia, L. J. (1997). *J. Appl. Cryst.* **30**, 565.

[bb3] Farrugia, L. J. (1999). *J. Appl. Cryst.* **32**, 837–838.

[bb4] Fedorov, B. S., Golovina, N. I., Fadeev, M. A., Strukov, G. V., Kedrov, V. V., Shilov, G. V., Boiko, G. N. & Atovmyan, L. O. (2001). *Russ. Chem. Bull.* **50**, 520–524.

[bb5] Jiao, J., Huang, Q., Cao, X., Li, Q. & Zhang, D. (1990). *Chin.* *J. Med. Chem.* **1**, 75–76.

[bb6] Nonius (2000). *COLLECT* Nonius BV, Delft, The Netherlands.

[bb7] Otwinowski, Z. & Minor, W. (1997). *Methods in Enzymology*, Vol. 276, *Macromolecular Crystallography*, Part A, edited by C. W. Carter Jr & R. M. Sweet, pp. 307–326. New York: Academic Press.

[bb8] Samejima, M. (1960). *Chem. Pharm. Bull.* **80**, 1706–1712.

[bb9] Sheldrick, G. M. (2008). *Acta Cryst.* A**64**, 112–122. 10.1107/S0108767307043930 18156677

